# Clinical and Antimicrobial Evaluation of *Copaifera langsdorffii* Desf. Dental Varnish in Children: A Clinical Study

**DOI:** 10.1155/2021/6647849

**Published:** 2021-03-25

**Authors:** Lídia Audrey Rocha Valadas, Patrícia Leal Dantas Lobo, Said Gonçalves da Cruz Fonseca, Francisco Vagnaldo Fechine, Edilson Martins Rodrigues Neto, Marta Maria de França Fonteles, Lorena Ribeiro de Aguiar Trévia, Hilda Lara Prado Vasconcelos, Sandra Mara da Silva Lima, Mara Assef Leitao Lotif, Analice Mendes Barreto Fernandes, Mary Anne Medeiros Bandeira

**Affiliations:** Federal University of Ceara, Fortaleza, Ceará, Brazil

## Abstract

**Background:**

The objective of this study was to evaluate the clinical and microbiological efficacies of (*C. langsdorffii*) dental varnish in children at high risk of dental caries..

**Methods:**

This is a longitudinal, randomized, controlled clinical trial. Ninety high-risk caries-free children (ICDAS II = 0) were recruited and randomly divided into three groups: *C. langsdorffii*, chlorhexidine, or fluoride. The varnishes were applied on the second deciduous molars for three times: baseline (D0), after 90 days (D90), and after 180 days (D180). Saliva was collected on D0, D90, D180, and D360 to evaluate *S. mutans* reduction. Statistics were carried out by ANOVA, Tukey's test, and the paired *t*-test.

**Results:**

Copaiba varnish demonstrated significant *S. mutans* reduction: D360 versus D0 (*p* < 0.0001), D180 versus D0 (*p* < 0.001), D360 versus D90 (*p* < 0.001), D180 versus D90 (*p* < 0.001), and D360 versus D180 (*p* < 0.05). Chlorhexidine varnish significantly reduced *S. mutans* at D180 versus D0 (*p* < 0.05). Fluoride reduced at D180 versus D0 (*p* < 0.001).

**Conclusions:**

Three annual applications of this varnish showed substantial antimicrobial activity against *S. mutans* and caries prevention for up to 12 months.

## 1. Introduction

Medicinal plants have great biological and pharmacological diversities, being great targets in drug development [1]. There are more than 100 million bioactive molecules cataloged, and this number can be considered unlimited due to still unexplored possible chemical arrangements and resources [2].

Copaiba tree belongs to the Leguminosae family, Caesalpinioideae subfamily, and *Copaifera* genus, and the trees can reach up to 400 years of age. The oilresin is yellow-brown with several active components such as sesquiterpenes and diterpenes, which have anti-inflammatory, analgesic, antimicrobial, and antitumor properties [3]. Copaiba oilresin has been widely used and especially found in neotropical regions where bees of the *Apis mellifera* species are the main pollinating agents. There are records of copaiba oilresin use for almost 400 years, with several studies proving its innumerable biological activities, being effective against several microorganisms and commonly used in traditional medicine against various diseases [3, 4].

The genus *Copaifera* is widely found in South and Central America, India, and West Africa. The greatest species richness is found in Brazil, where trees can especially be found in the Southeast, Midwest, and Amazon regions. Among the 72 cataloged species, 20 have already been found in Brazil, with 16 being exclusive to the country and considered a food, thus presenting its safety proven by the wide traditional popular use. Among them are *Copaifera officinalis* L., *Copaifera guianensis* Desf., *Copaifera reticulata* Ducke, *Copaifera multijuga* Hayne, *Copaifera confertiflora* Benth., *Copaifera langsdorffii* Desf. (*C. langsdorffii*), *Copaifera coriacea* Mart., and *Copaifera cearensis* [3, 5, 6].

*C. langsdorffii* oilresin is effective against Gram-positive and Gram-negative bacteria, especially for topical use. Its effectiveness against the cariogenic bacterial is also emphasized [7]. Copaiba oilresin has high activity against oral bacteria and can be used in appropriate formulations, since the main oral diseases, caries, and periodontal diseases are strongly related to the dental biofilm. However, in vitro and in vivo assays of these formulations must be well studied [5].

Dental caries is the most prevalent disease in the world and is called early childhood caries (ECC) when it occurs in children under 6 years of age. This condition specially affects the deciduous second molars due to their occlusal morphology which favors accumulating bacterial plaque [8, 9]. Even with the expansion of access to health services, early childhood caries (ECC) is still a public health problem [10], being the main cause of losing deciduous teeth early, negatively influencing speech, aesthetics, the masticatory system, and the dental arches [11].

The main products in preventing oral diseases are fluoride, chlorhexidine, triclosan, cetylpyridinium chloride, and natural products, especially extracts and essential oils which have attracted attention due to antimicrobial activity. These have also been used as therapeutic alternatives against dental caries [12–14].

Dental biofilm is the main biological determinant in the development of dental caries, and salivary microbiota is related to tooth decay [15, 16]. Several microorganisms colonize dental biofilms, where *S. mutans* are strongly associated with dental caries, and are found in all niches such as saliva, tongue, oral mucosa, and dental plaque [17]. After consumption of sucrose, *S. mutans* produce extracellular polysaccharides and are acidogenic and aciduric, being able to survive in adverse conditions. The oral cavity presents several genotypes of *Streptococcus mutans* (*S. mutans*) with different virulence capacities [10, 18]. Thus, although they are not the only ones involved in the process, they are a key contributor in forming dental biofilms and can be considered a salivary biomarker [19, 20].

Healthy behavioral approaches and promotion should be implemented for the prevention of dental caries in public health, in addition to policies such as public water fluoridation and strategies in high-risk groups with restricted access to dental and fluoride services [9]. It is also known that preventing decay in primary teeth will prevent permanent dentition. In the current growing model of minimally invasive dentistry, it is argued that dental caries can be controlled and prevented in a noninvasive way through several products, among which varnishes can be mentioned [17, 21].

As children under six years of age do not yet have the proper habit of “rinsing and spitting,” the most appropriate formulations for preventing caries disease at this age would be varnishes rather than a rinse aid or gel and also because of their high retentive capacity and the slow release of the active principle.

No reports have been found in the literature on the use of varnishes based on copaiba, and thus, the application of a patent of invention was deposited under protocol BR 1020160212628. The objective of this study was to evaluate the clinical and microbiological efficacies of *C. langsdorffii* dental varnish in children at high risk of caries.

## 2. Materials and Methods

### 2.1. Copaiba Oilresin

Samples of copaiba oilresin obtained from *Copaifera langsdorffii* Desf. (Fabaceae: Caesalpinioideae) plant material deposited in the herbarium of the Federal University of Mato Grosso, voucher Silva, R. R. et al. 1749, were received from the Federal University of Mato Grosso and originally obtained from Juruena Valle (region: midwest, latitude: 10° 19' 05” S, longitude: 58° 21' 32” W, and height: 300 m). Chemical constituents were identified by specialists at the Department of Chemistry in the Federal University of Ceara (GC–MS QP 5050, Shimadzu, Japan). The total content of the bioactive constituents was 84.69%. The main compounds were caryophylene oxide (54.2%), *β*-caryophylene (6.08%), *β*-element (4.43%), *α*-cis-bergamotene (4.56%), and ar-curcumene (4.63%).

### 2.2. Clinical Study

This is a longitudinal, parallel, randomized, double-blind controlled clinical trial. The rules of the CONSORT checklist were followed in order to improve the study methodology.

### 2.3. Local and Population/Ethical Aspects

This study was approved by the Ethics Committee of the Federal University of Ceara (UFC), with number 195.096. The clinical phase occurred in the city of Aracati-CE-Brazil, a city in which only 0.8% of the population has fluoridated public water coverage. The parents were invited to participate in their search and then informed so as to sign the clear and informed consent form. The population selection (90 children) was carried out by means of a clinical examination of the patients in public schools and daycare centers, where children who were free of caries (ICDAS II 0) with 4 erupted primary second molars, aged between 36 and 71 months and of both genders, were included. The detection was performed by a single researcher calibrated for ICDAS II (kappa index 0.78). The high-risk caries classification was performed according to the criteria of the American Academy of Pediatric Dentistry (AAPD, 2014) [22], for example, consumption of sugar more than three times a day, lack of access to fluoridated water, presence of visible plaque, poor oral hygiene, and absence of visits to a professional dentist. Exclusion criteria were presence of any buccalor systemic disease or the application or use of any antibiotic or antimicrobial three months prior to starting the study.

### 2.4. Varnish Preparation

Copaiba oilresin was formulated as a varnish in the pharmacotechnical laboratory of the pharmacy course of the Faculty of Pharmacy, Dentistry, and Nursing of the Federal University of Ceara, in a standardized way in order to obtain similarity of color, odor, consistency, and flavour.

A pilot study was initially performed to obtain the dose-response curve [23]. The concentration (1%) used in the main study was first checked as having the greatest relative reduction capacity of bacteria (%). All varnishes were stored in tubes and encoded with letters of the alphabet to ensure blinding in this study.

### 2.5. Grouping, Application of Varnishes, and Saliva Collection

After randomization in the Excel program, children were divided into 3 groups, with 30 participants each. The sample for each group was calculated considering a power of 90% and a significance level of 5%. The sample size needed to satisfy the requirements of this study was calculated as being 24 subjects in each group. However, 25% was added to this value in order to cover possible follow-up losses; then, the final sample size was estimated as 30 patients in each group. All participants received a toothbrush of the same brand with a straight handle, small head, and soft bristles and fluoridated toothpaste to use thrice a day.

Group I received application of 1% chlorhexidine varnish, 5% fluoride varnish group II, and 1% *C. langsdorffii* oilresin group (copaiba). Each patient initially chewed a piece of 3 × 3 cm plastic film (Parafilm^®^) for 60 s to stimulate the production of saliva and release the bacteria from the dental biofilm. All participants received the same type of toothbrush and fluoridated toothpaste. Standardized oral hygiene instruction was conducted through a single instructor for all parents that received the recommendations to be followed in writing to reinforce the instructions.

Saliva was collected using a plastic device and stored in sterile microcentrifuge tubes (Eppendorf^®^), which were stored in polystyrene box containing ice. To minimize the influence of the circadian rhythms on salivary flow, all samples were collected in the same session and conditions by the same operator between 9 : 00 and 11 : 00 AM. After the collection of saliva, each patient received an application of the varnish corresponding to their group in the four second deciduous molars. Prior to an application of varnishes, the teeth were professionally cleaned with a Robinson brush and pumice. The varnishes were applied with relative insulation on to the selected molars using a microbrush. After 10 s, the varnish was subtly dried by air from a triple syringe. The cotton rolls were removed after 25 seconds to avoid saliva contamination. The varnish was applied 3 times for each tooth: at the baseline, after 90 days, and after 180 days of starting treatment. The presence or absence of caries was also recorded in the evaluated teeth as well as in others during each evaluation and after the baseline. The saliva of each patient was collected at 4 moments: at the baseline, after 90 days, after 180 days, and after 360 days of starting treatment.

### 2.6. Microbiological Analysis

Samples were transported to the laboratory for microbiological analysis in a hermetically sealed case containing ice.

Saliva was homogenized on a tube shaker for 30 seconds. A volume of 0.1 mL of each sample was aseptically drawn and transferred into one sterile test tube containing 0.9 mL of saline. The procedure was repeated twice, establishing dilutions of 1 : 10 and 1 : 100. A corresponding volume of 10 *μ*L of each dilution was plated onto mitis salivarius-bacitracin (MSB) agar medium in triplicates. The plates were then incubated at 37°C during 48 h in jars under microaerophilic conditions. Bacterial counts were expressed as colony forming units (CFU)/mL of saliva and followed by phenotypical colony identification, as described elsewhere.

### 2.7. Clinical Evaluation

The children were evaluated for monitoring carious lesions by the ICDAS II method in the same saliva collection periods.

### 2.8. Statistical Analysis

The data regarding the number of CFU were initially transformed in order to achieve normal distribution, using a logarithmic transformation (log10). The transformed values of the CFU number were initially analyzed by the Kolmogorov–Smirnov test to verify the normality of the distribution. Thus, mean and standard deviation were calculated for the descriptive statistics, as well as parametric tests were used for data analysis. Analysis of variance (ANOVA) was used to compare the three groups at each time (intergroup analysis), associated with Tukey's multiple comparisons test to verify differences between the paired groups. Comparisons between the different times within each group (intragroup analysis) were performed by repeated measures analysis of variance (ANOVA), associated with Tukey's multiple comparisons test in order to verify differences between paired times. The level of significance was set at 0.05 (5%) in all analyzes, with a *p* value less than 0.05 being considered as statistically significant. GraphPad Prism® software version 5.00 for Windows® (GraphPad Software, San Diego, California, USA, 2007) was used for both statistical procedures and graphing.

## 3. Results

**Figure 1:**
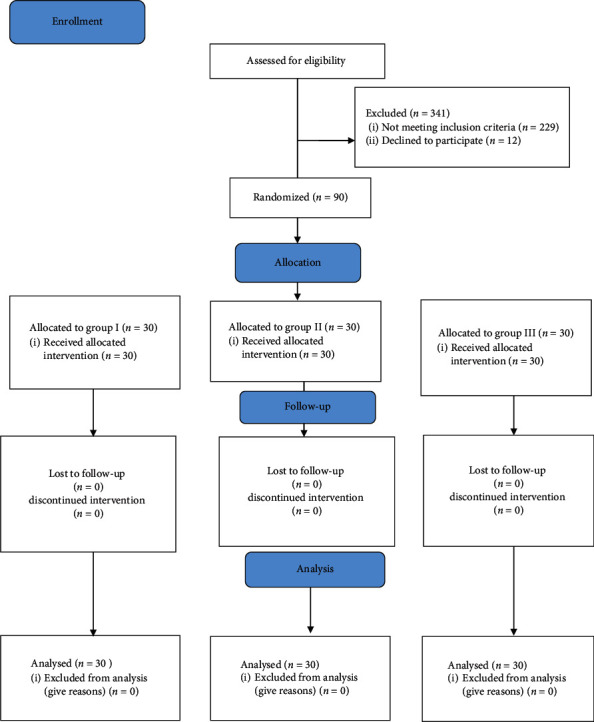
CONSORT 2010 flow diagram of the study.

We have randomized 90 children in three groups (Figure 1). All the participants completed the study.

Table 1 shows the amount of *S. mutans*, expressed as the logarithm of the number of colony forming units (CFU) per mL of saliva, measured in saliva samples with dilution of 1 : 10 on days 0 (baseline), 90, 180, and 360 in patients treated with chlorhexidine, fluoride, and copaiba varnishes on the first dilution. The data correspond to the mean and standard deviation of the logarithm of the number of CFU verified in the saliva samples of the patients in each treatment group. At the end of treatment, the groups treated with copaiba varnish (*p* < 0.0001) and fluoride (*p* < 0.0001) had higher statistical difference in relation to the start of treatment compared with the chlorhexidine group (*p*=0.0107).

Table 2 shows the amount of *S. mutans*, expressed as the logarithm of the number of colony forming units (CFU) per ml of saliva, measured in saliva samples with dilution of 1 : 100 on days 0 (pretreatment), 90, 180, and 360 in patients treated with chlorhexidine, fluoride, and copaiba varnishes, on the second dilution (1 : 100). The data correspond to the mean and standard deviation of the logarithm of the number of CFU verified in the saliva samples of the patients in each treatment group. At the end of treatment, the group treated with chlorhexidine (*p* < 0.05) and copaiba (*p* < 0.001) varnishes had a statistical difference in relation to D0.

Regarding the clinical data at the end of the groups with different treatment of dental varnishes (Table 3), the appearance of 5 initial carious lesions in the group treated by the chlorhexidine varnish was observed in 2 patients. The group treated with copaiba and fluoride varnishes registered no caries lesion using ICDAS II scores.

## 4. Discussion

In the present study, the clinical and antimicrobial efficacies of a new varnish containing copaiba oilresin were evaluated along 360 days and compared with dental varnishes with fluoride and chlorhexidine, in order to prevent dental caries in a high-risk group of children.

Among the risk markers for ECC are the *S. mutans* and *Lactobacillus* species, which are part of the oral microbiome [22]. These may reflect different stages of the caries process and reveal changes in the oral microbiota [15]. Although several species are involved in dental caries, *S. mutans* are still strongly associated with the disease; its high colonization in the oral cavity may be associated with the disease, since they are an indicator of microbial disequilibrium [12, 13, 24]. Streptococci, although not the only ones involved in dental caries, are one of the major colonizers of the oral cavity, initiating this colonization soon after tooth eruption [17]. Saliva is a representative medium of the oral microbiota, which may reflect the changes in it, and was chosen because it is an accessible medium [15].

Studies evaluating the use of chlorhexidine for a period of 6 months were insufficient to verify the effect on dental caries, as most do not show any effect on disease control [25]. Vale et al. (2014) [26] evaluated the time of recolonization of *S. mutans* after two consecutive days of treatment with 1% chlorhexidine gel. Saliva was collected before the study and at days 1, 7, 14, 21, and 28 for evaluation of *S. mutans* levels. The levels decreased but were not statistically significant. In this present study, the chlorhexidine varnish reduced the CFU for a period of six months (*p*=0.0107), but in the last analysis (D360), it was observed that the CFU returned to the same levels of the initial period.

The topical use of fluoride products in high concentrations (>2,500 ppm) creates fluoride reservoirs, providing fluoride to the dental surface and promotes its penetration into the biofilm, being effective in reducing demineralization and increasing remineralization. Fluoride may present bactericidal activity with frequent professional applications. The group treated with fluoride varnish in the present study showed a reduction of CFU throughout the study period (*p* < 0.0001).

The antimicrobial activity of copaiba oilresin may be related to the combination of sesquiterpenes and diterpenes, thus affecting the integrity of the bacterial cell wall. The oil has scientifically proven activity against several pathogens, especially Gram-positive bacteria such as *Staphylococcus* spp. and *Streptococcus* spp. It is important to use a suitable methodology for the dilution of oilresin in research and validation by gas chromatography [3]. In the present study, copaiba showed a significant reduction of CFU throughout all the periods (*p* < 0.0001). In the intergroup analysis in each period studied, the group treated with copaiba varnish was the only one to show statistically significant results for the two dilutions.

According to Diefenbach et al. (2018) [5], most of the studies which evaluate the antimicrobial activity of copaiba oilresin compared it with chlorhexidine, which is the positive control, where *S. mutans* are the most studied organisms, as well as the other studies with natural products in Dentistry [14].

Pieri et al. (2010) [27] evaluated the action of *β*-caryophyllene isolated from copaiba oilresin on the adhesion of *S. mutans* bacteria, in which it had better action than chlorhexidine. Pieri et al. (2016) [28] evaluated the antimicrobial activity of *β*-caryophyllene isolated from copaiba oilresin against dental plaque bacteria in vitro. The results demonstrated that *β*-caryophyllene prevented plaque-forming bacteria from proliferating.

Dental varnishes stand out in preventing dental caries and are widely accepted by pediatric patients, especially children under 6 years of age. Patients in this age group do not have adequate capacity to eject saliva, so the varnishes were the chosen formulations for the use of copaiba oilresin [29, 30]. They are composed of polymer matrices, excipients, and active principle. In the case of the present varnish, the chosen matrix was insoluble (in this case, ethylcellulose), used to modulate the release of the active principle, and thus, its substantivity was higher [21, 23]. This type of formulation adheres to dental scars and fissures, gradually releasing the active principle, disrupting the dental biofilm, and becoming a long-term therapeutic agent which is suitable for antimicrobial formulation [29, 31].

Most of the randomized clinical trials with outcomes in dental caries are currently focused on the performance of restorative materials and with many biases in the sample. In the current phase of minimally invasive dentistry, studies with materials and preventive alternatives are important [32].

In the pilot study of copaiba dental varnish, all oilresin concentrations showed antimicrobial activity; however, only 1% showed a reduction in *S. mutans* colony forming units [23]. It is believed that the greater complexity of the chemical constituents present in copaiba, a pharmaceutical form with lower concentration, presents a smaller interaction between the pharmaceutical excipients used in the formulation. In addition, the active ingredients of copaiba were probably retained in the varnish matrix and were released locally. It was also observed that higher concentrations of copaiba oilresin lost the ability to retain its active principle, and in these situations, the active principle was released so quickly that the varnish partially lost its antimicrobial activity.

For clinical applications, copaiba and fluoride varnishes showed similar results in preventing dental caries. However, it is important to consider that it is a study with small sample and only one year of follow-up was considered.

## 5. Conclusions

After three annual applications, copaiba varnish demonstrated significant antimicrobial activity against *S. mutans* for up to 12 months in children with high risk of caries. The fluoride and copaiba varnishes had good results regarding dental caries prevention. Future studies are needed to identify anticaries effects to establish the use of varnish in caries prevention.

## Figures and Tables

**Table 1:** Amount of *S. mutans*, expressed as the logarithm of the number of colony forming units (CFU) per mL of saliva, measured in saliva samples with dilution of 1 : 10 on days 0 (pretreatment), 90, 180, and 360 in patients treated with chlorhexidine, fluoride, and copaiba varnishes.

	Chlorhexidine	Fluoride	Copaiba	Significance (ANOVA)
	Mean ± SD	Mean ± SD	Mean ± SD	
0	0.58 ± 0.43	0.86 ± 0.37	1.32 ± 0.61^a,d^	
90	0.38 ± 0.23	0.51 ± 0.33^x^	0.99 ± 0.57^a,c,y^	
180	0.33 ± 0.14^y^	0.41 ± 0.24^x^	0.39 ± 0.22^x,z^	*P*=0.3580
360	0.55 ± 0.52	0.53 ± 0.44^x^	0.12 ± 0.19^b,d,x,z^	
Significance (repeated measures ANOVA)				—

SD, standard deviation; ANOVA, analysis of variance. The letters ^a^(*P* < 0.001) and ^b^(*P* < 0.01) denote statistically significant differences in relation to the chlorhexidine varnish on the same day, while the letters ^c^(*P* < 0.001) and ^d^(*P* < 0.01) indicate statistically significant differences in relation to the fluoride varnish on the same day (Tukey test). The letters ^x^(*P* < 0.001) and ^y^(*P* < 0.05) designate statistically significant differences in relation to day 0 in the same group, while the letter ^z^(*P* < 0.001) denotes statistically significant difference in relation to day 90 in the same group (Tukey's test).

**Table 2:** Amount of *Streptococcus mutans*, expressed as the logarithm of the number of CFU per mL of saliva, measured in saliva samples with dilution of 1 : 100 on days 0 (pretreatment), 90, 180, and 360 in patients treated with chlorhexidine, fluoride, and copaiba varnishes.

Day	Chlorhexidine	Fluoride	Copaiba	Significance (ANOVA)
	Mean ± SD	Mean ± SD	Mean ± SD	
0	0.45 ± 0.26	0.48 ± 0.20	0.89 ± 0.55^a.c^	
90	0.33 ± 0.13	0.36 ± 0.11	0.66 ± 0.44^a.c^	
180	0.30 ± 0.00^v^	0.34 ± 0.07^w^	0.38 ± 0.19^u.y^	*P*=0.0723
360	0.31 ± 0.18^w^	0.40 ± 0.28	0.10 ± 0.19^b.c.u.x.z^	
Significance (repeated measures ANOVA)				—

SD, standard deviation; ANOVA, analysis of variance. The letters ^a^(*P* < 0.001) and ^b^(*P* < 0.01) denote statistically significant differences in relation to the chlorhexidine varnish on the same day, while the letter ^c^(*P* < 0.001) indicates statistically significant difference in relation to the fluoride varnish on the same day (Tukey test). The letters ^u^(*P* < 0.001), ^v^(*P* < 0.01), and ^w^(*P* < 0.05) designate statistically significant differences in relation to day 0 in the same group; the letters ^x^(*P* < 0.001) and ^y^(*P* < 0.05) denote statistically significant differences in relation to day 90 in the same group, while the letter ^z^(*P* < 0.05) indicates statistically significant difference in relation to day 180 in the same group (Tukey test).

**Table 3:** Distribution of the lesions on the molars (scores ICDAS II) of different groups treated with dental varnishes at the end of the study.

Tooth	Copaiba	Chlorhexidine	Fluoride
Score 0	0	0	0
Score 1	0	4	0
Score 2	0	1	0
Score 3	0	0	0
Score 4	0	0	0
Score 5	0	0	0
Score 6	0	0	0

## Data Availability

The data used to support the findings of this study are available at the repository http://repositorio.ufc.br/bitstream/riufc/44474/1/2019_tese_larvmarques.pdf.
